# Efficacy of levocetirizine for the treatment of children with allergic rhinitis: A protocol for systematic review and meta-analysis

**DOI:** 10.1097/MD.0000000000020557

**Published:** 2020-06-05

**Authors:** Peng-ju Zheng, Jin-sheng Wang, Gui-fang Liu, Shu-hua Zhang, Yi-ying Zhang

**Affiliations:** a Department of Otolaryngology, First Affiliated Hospital of Jiamusi University; b Department of Otorhinolaryngology, Second Hospital of Jiamusi Agricultural Reclamation; c Department of Otolaryngology, Jiamusi University Affiliated Stomatological Hospital, Jiamusi, China.

**Keywords:** allergic rhinitis, children, efficacy, levocetirizine, safety

## Abstract

**Background:**

Although previous studies have reported that levocetirizine is utilized for the treatment of children with allergic rhinitis (AR), its conclusions remain inconsistent. This study aims to evaluate the efficacy and harms of levocetirizine for children with AR.

**Methods:**

Electronic database sources will be undertaken from the beginning to the present: MEDLINE, EMBASE, The Cochrane Library, CINAHL, ACMD, PsycINFO, Chinese Biomedical Literature Database, and China National Knowledge Infrastructure. We will not apply any restrictions to language and publication status. We will only consider randomized controlled trials of levocetirizine for children with AR. Two authors will independently scan literature, select studies, and collect data. Study quality for each included trial will be assessed using Cochrane risk of bias tool, and statistical analysis will be conducted using RevMan 5.3 software.

**Results:**

This study will summarize the present evidence to systematically assess the efficacy and harms of levocetirizine for children with AR.

**Conclusion:**

The findings of this study intent to adequately inform stakeholders or clinicians, as well as to help develop treatment guidelines.

**Study registration number:**

INPLASY202040111.

## 1 Introduction

Allergic rhinitis (AR) is a global health problem, which involves an inflammatory process in nasal mucosa.^[1–3]^ It is estimated that about 10% to 25% people affect this condition worldwide.^[4–5]^ The main symptoms of AR comprises of sneezing, watery eyes, and nasal discharge, burning, itching, and obstruction.^[6–7]^ These symptoms have a remarkable toll on quality of life in such patients.^[8–9]^

Levocetirizine is a potent second-generation histamine receptor antagonist.^[10]^ It is reported to effectively against children with AR and to improve the quality of life.^[4,11–19]^ However, there is not systematic literature available regarding the efficacy and harms of levocetirizine for children with AR. Thus, this study aims to compare the efficacy and safety of levocetirizine with other modalities for the treatment of children with AR.

## 2 Methods and analysis

### 2.1 Study registration

The current protocol has been registered on INPLASY202040111. We have reported it based on the guidelines of Preferred Reporting Items for Systematic review and Meta-Analysis Protocols.^[20–21]^

### 2.2 Eligibility criteria

#### 2.2.1 Type of studies

Types of studies are randomized controlled trials on investigating the efficacy and harms of levocetirizine for children with AR regardless their publication type, publication time and language. We will not consider any other studies, such as reviews, case studies.

#### 2.2.2 Type of participants

Any children (below 18 years old) diagnosed with AR will be included regardless their country, race, gender, and economic background.

#### 2.2.3 Type of interventions

We will accept any forms of levocetirizine as an interventional treatment in the experimental group. However, we will remove studies with combination of levocetirizine and other modalities.

In the control group, we accept any treatments, except any types of levocetirizine, including its single or combination modes.

#### 2.2.4 Type of outcomes

The primary outcome is total nasal symptoms. It consists of nasal symptoms (sneezing, runny nose, nasal itching, and nasal congestion) and ocular symptoms (eye itching, foreign body sensation, red eyes, tearing). It can be measured by any appropriate scales or other forms of tools, such as the Total Nasal Symptom Score.

The secondary outcomes are quality of life (as identified by any scores, such as the Rhinoconjunctivitis Quality of Life Questionnaire), global non-nasal symptoms (as assessed by any validated daily or weekly diaries or scores, such as visual analogue scales), use of conventional medication (as evaluated by Medication Quantification Scale or any other scales), laboratory indicators, and any expected or unexpected adverse events.

### 2.3 Search strategy

We will carry out a comprehensive search including MEDLINE, EMBASE, The Cochrane Library, CINAHL, ACMD, PsycINFO, Chinese Biomedical Literature Database, and China National Knowledge Infrastructure from their beginning to the present without restrictions of language and publication status. The detailed search strategy for MEDLINE is created by a professional librarian (Table 1). We will adapt similar search strategies to the other electronic databases. Further searches will be conducted for abstracts of scientific conferences/symposia, or reference lists of relevant reviews or clinical trial registries for ongoing trials.

**Table 1:**
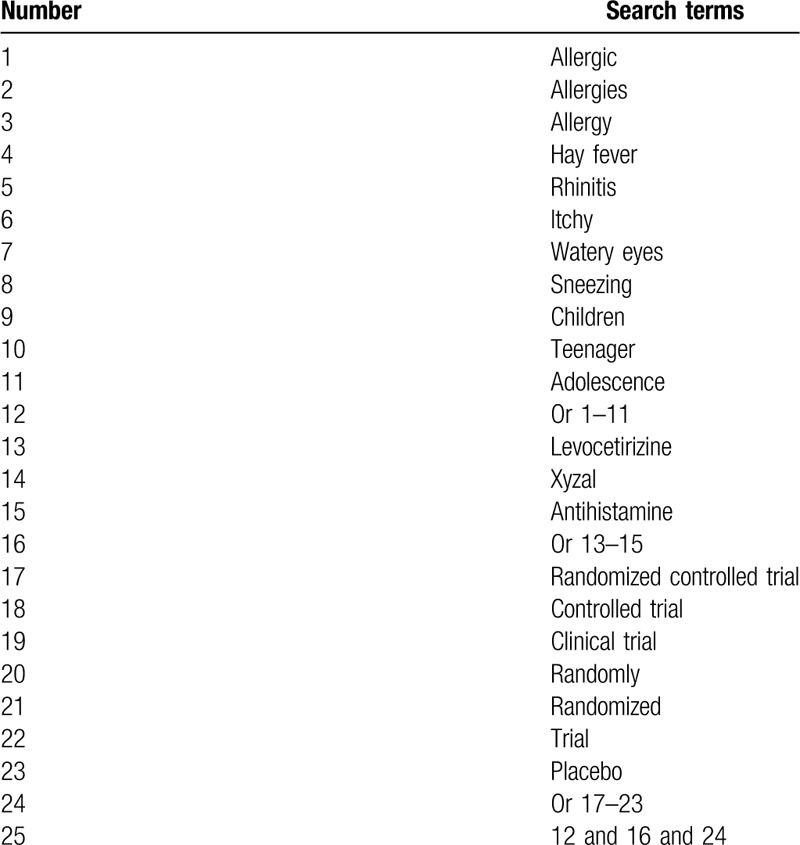
Search strategy applied in MEDLINE.

### 2.4 Data collection and management

#### 2.4.1 Study selection

Two authors will independently scan titles and abstracts for all citations, and any unrelated records will be removed from this stage. At the next stage, we will obtain full-text of all remaining studies to identify trials against inclusion criteria, and will note reasons for exclusion of the unqualified studies. We will solve any divergences through discussion by consultation a third experienced author. We will present the selection process in detailed information in a flowchart.

#### 2.4.2 Data extraction

Two authors will independently extract data from included trials using data collection sheet, which has been piloted on at least 2 trials. Any inconsistent views will be solved by involving a third experienced author. We will collect the following information:

Trial characteristics: title, first author, year of publication, et al;
Patient characteristics: race, gender, age, diagnostic criteria, inclusion and exclusion criteria, number of patients, et al;
Methods: trial design, trial setting, details of randomization, blind, et al;
Interventions and controls: delivery modes, dosage, frequency, duration, et al;
Outcomes: primary and secondary outcomes, any expected and unexpected adverse events, et al;
Others: funding for trial, conflict of interests of study authors.

If we identified any insufficient or missing information, we will contact original authors to obtain them. If these data are not available, we will only utilize the available data for statistical analysis. Additionally, we will also discuss its potential impacts as limitation in the manuscript.

### 2.5 Assessment of risk of bias for included trials

Two authors will independently evaluate the risk of bias for each eligible trial using Cochrane risk of bias tool. It covers aspects of allocation sequence generation, allocation concealment, blinding of participants and treatment providers, blinding of outcome assessment, incomplete outcome data, selective outcome reporting, and other bias. Each aspect is further judged as low, unclear or high risk of bias. In case of disagreements, a third author will help resolve them by consultation.

### 2.6 Statistical analysis

We will use RevMan 5.3 Software for data synthesis and statistical analysis.

#### 2.6.1 Data synthesis

For continuous data, we will calculate it using standardized mean difference and 95% confidence intervals. For dichotomous data, we will elaborate it using risk ratio and 95% confidence intervals. We will assess the presence of statistical heterogeneity using *I*^2^ statistic test and will interpret its values as follows: *I*^2^ ≤50% exerts acceptable heterogeneity, while *I*^2^ > 50% presents significant heterogeneity. If *I*^2^ ≤50%, we will use a fixed-effects model. If sufficient data are obtained, we will synthesize the data and will conduct a meta-analysis. Otherwise, if *I*^2^ > 50%, we will apply a random-effects model. We will carry out subgroup analysis to explore the possible reasons for the substantial analysis.

#### 2.6.2 Subgroup analysis

We will undertake subgroup analysis based on the different types of interventions and controls, characteristics of study or patient, and different outcome measurements.

#### 2.6.3 Sensitivity analysis

In the case of sufficient data, we will carry out sensitivity analysis to check the robustness of pooled outcome results based on the study characteristics, or methodological quality by excluding high risk of bias trials.

#### 2.6.4 Reporting bias

Funnel plot and Egger regression test will be utilized to check the reporting biases when the number of eligible trials entered in a meta-analysis is over 10.^[22–23]^

### 2.7 Quality of evidence

The quality of evidence of each outcome will be assessed through Grading of Recommendations Assessment Development and Evaluation.^[24]^ It covers 5 domains and each 1 will be graded as high, moderate, low, or very low based on the Grading of Recommendations Assessment Development and Evaluation rating standards.

### 2.8 Ethics and dissemination

In this study, we do not need ethical approval, because no individual patient data will be obtained. This study is expected to be published on a peer-reviewed journal or conference meeting.

### 2.9 Patient and public involvement

Patients and/or the public were not directly involved in the development of this study protocol.

## 3 Discussion

Previous studies have reported the efficacy and harms of levocetirizine for the treatment of children with AR.^[4,11–19]^ However, there is a gap of efficacy and safety between levocetirizine and children with AR at literature level. Thus, the goal of this study is to bridge the gap in this field. It will summarize the evidence regarding the efficacy and harms of levocetirizine for the treatment of children with AR. It will provide insight on the extent to clinician and health-related policy maker.

## Acknowledgment

This study is supported by the Heilongjiang Provincial Health and Family Planning Commission Research Project (2014–246). The funders had no roles, and no conflict interests with this study.

## Author contributions

**Conceptualization:** Peng-ju Zheng, Jin-sheng Wang, Gui-fang Liu, Yi-ying Zhang.

**Formal analysis:** Peng-ju Zheng, Jin-sheng Wang, Shu-hua Zhang, Yi-ying Zhang.

**Funding acquisition:** Yi-ying Zhang.

**Investigation:** Yi-ying Zhang.

**Methodology:** Peng-ju Zheng, Jin-sheng Wang, Gui-fang Liu, Shu-hua Zhang.

**Project administration:** Yi-ying Zhang.

**Resources:** Peng-ju Zheng, Jin-sheng Wang, Gui-fang Liu, Shu-hua Zhang.

**Software:** Peng-ju Zheng, Jin-sheng Wang, Gui-fang Liu, Shu-hua Zhang.

**Supervision:** Yi-ying Zhang.

**Validation:** Peng-ju Zheng, Jin-sheng Wang, Yi-ying Zhang.

**Visualization:** Peng-ju Zheng, Gui-fang Liu, Shu-hua Zhang, Yi-ying Zhang.

**Writing – original draft:** Peng-ju Zheng, Jin-sheng Wang, Gui-fang Liu, Shu-hua Zhang, Yi-ying Zhang.

**Writing – review and editing:** Peng-ju Zheng, Gui-fang Liu, Shu-hua Zhang, Yi-ying Zhang.
